# Antigenicity and adhesiveness of a *Plasmodium vivax* VIR-E protein from Brazilian isolates

**DOI:** 10.1590/0074-02760210227

**Published:** 2022-02-04

**Authors:** Ana Paula Schappo, Najara C Bittencourt, Leticia P Bertolla, Sofia Forcellini, Ana Beatriz Iung Enembreck da Silva, Hellen Geremias dos Santos, João Henrique Gervásio, Marcus VG Lacerda, Stefanie CP Lopes, Fabio TM Costa, Letusa Albrecht

**Affiliations:** 1 Fundação Oswaldo Cruz-Fiocruz, Instituto Carlos Chagas, Laboratório de Pesquisa em Apicomplexa, Curitiba, PR, Brasil; 2 Universidade Estadual de Campinas, Instituto de Biologia, Departamento de Genética, Evolução, Microbiologia e Imunologia, Campinas, SP, Brasil; 3 Fundação Oswaldo Cruz-Fiocruz, Instituto Leônidas & Maria Deane, Manaus, AM, Brasil; 4 Fundação de Medicina Tropical Dr Heitor Vieira Dourado, Gerência de Malária, Manaus, AM, Brasil

**Keywords:** Plasmodium vivax, multigene family, antigenicity, adhesiveness

## Abstract

**BACKGROUND:**

*Plasmodium vivax*, the major cause of malaria in Latin America, has a large subtelomeric multigene family called *vir*. In the *P. vivax* genome, about 20% of its sequences are *vir* genes. *Vir* antigens are grouped in subfamilies according to their sequence similarities and have been shown to have distinct roles and subcellular locations. However, little is known about *vir* subfamilies, especially when comes to their functions.

**OBJECTIVE:**

To evaluate the diversity, antigenicity, and adhesiveness of *Plasmodium vivax* VIR-E.

**METHODS:**

*Vir*-E genes were amplified from six *P. vivax* isolates from Manaus, North of Brazil. The presence of naturally acquired antibodies to recombinant PvBrVIR-E and PvAMA-1 was evaluated by ELISA. Binding capacity of recombinant PvBrVIR-E was assessed by adhesion assay to CHO-ICAM1 cells.

**FINDINGS:**

Despite *vir*-E sequence diversity, among those identified sequences, a representative one was chosen to be expressed as recombinant protein. The presence of IgM or IgG antibodies to PvBrVIR-E was detected in 23.75% of the study population while the presence of IgG antibodies to PvAMA-1 antigen was 66.25% in the same population. PvBrVIR-E was adhesive to CHO-ICAM1.

**MAIN CONCLUSIONS:**

PvBrVIR-E was antigenic and adhesive to CHO-ICAM1.

Malaria is the most disseminated human disease in the world, affecting more than 200 million people every year.^1^ Even though malaria rates have been reducing, *Plasmodium falciparum* remains the largest target of studies, while other *Plasmodium* species are still neglected.^2^ Capable to reach beyond Africa and also present in countries in Asia, Oceania and Americas, *P. vivax* predominates in regions with high population density.^3^ This parasite is responsible for more than 70% of the cases in the Americas and stands as the major cause of malaria in Brazil.^1^

Unlike the benign reputation of this parasite, *P. vivax* has the capacity to cause severe diseases.^4^ ^,^ ^5^ While in falciparum malaria the pathogenesis is associated to the parasite ability to adhere to endothelial cells and to non-infected erythrocytes (forming rosettes)^6^ in vivax malaria little is known about parasite cytoadherence.^7^ ^,^ ^8^ Variant surface antigens like *Plasmodium falciparum* erythrocyte membrane protein 1 (PfEMP1) and RIFIN codified by *var* and *rif* genes respectively, are responsible for the *P. falciparum* cytoadherence and linked to parasite ability of escaping from the immune system.^9^ ^,^ ^10^ While these proteins are absent in *P. vivax* another multigene family has been implicated in adhesion and antigenic variation. The *P. vivax* variant genes (*vir*) family is the largest multifamily of *P. vivax*. Initially, 346 *vir* genes were identified in *P. vivax* genome, with most of them located at subtelomeric regions and grouped in 12 subfamilies.^11^ Later, more than a 1000 *vir* genes were identified in *P. vivax* genomes.^12^ In fact, a previous work from del Portillo and colleagues suggest that 10-20% of *P. vivax* haploid genome are composed of *vir* genes.^13^ This vast diversity of VIR antigens allows the parasite to evade from the host immune system.^14^

VIR proteins can have distinct cellular locations which could imply in different functions.^15^ As part of the large *vir* family, the subfamily E presents a conserved N-terminal domain and polymorphic regions in the C-terminal.^16^ Displaying high diversity and being one of the most polymorphic subfamilies, Pexel-like motif are found in 100% of these sequences, suggesting that these proteins are expressed at the surface of infected reticulocytes.^16^ Interested in the great potential of *vir* genes as a central whole of *P. vivax* pathogenesis, the present study evaluated the genetic diversity, antigenicity and adhesiveness of a VIR-E protein.

## MATERIALS AND METHODS

*Blood sample collection* - Blood samples were collected from patients with malaria who were seeking medical care from 2012 to 2014 at Fundação de Medicina Tropical Dr Heitor Vieira Dourado, Manaus, Amazonas State, a low transmission region located in northern Brazil. Blood samples were collected using BD Vacutainer with sodium citrate anticoagulant. The blood was analysed just after its collection using a Sysmex KX21N (Sysmex Corporation-Roche, Japan). A thin blood smear was prepared from each blood sample to determine species of malaria parasites. The samples were confirmed *P. vivax* monospecies infection by Nested-PCR.^17^

*Amplification and sequencing of vir-e genes* - Genomic DNA from whole blood of six patients with vivax malaria was extracted using the phenol:chloroform method.^18^ *Vir* genes from subfamily E (*vir-E*) were amplified by polymerase chain reaction (PCR) using degenerate *vir*-E primers (forward 5′- AA(Y)CAAGAA(W)TTTAT(S)AACTTTGT-3′ and reverse 5′- TACC(Y)TATATA(W)CGTTATTAGAGG-3′) as previously described by Fernandes-Becerra et al.,^14^ using Platinum Taq DNA Polymerase High Fidelity (Thermo Fisher Scientific). PCR products were size separated by electrophoresis on a 1% agarose gel stained with ethidium bromide and visualised under a UV light and purified using *Wizard* ^*®*^ *SV Gel and PCR Clean-Up System* (Promega). Each amplified sequence was inserted into pGEM-T Easy Vector (Promega). Sequencing reactions were performed with T7 and SP6 primers and plasmids were sequenced using ABI 3100 (Applied Biosystems, CA, USA).

*Sequence analysis* - The quality of all acquired sequences was checked by Phred quality above 20 in a sliding window of 300 bp.^19^ Plasmid sequences were trimmed out and the sequences were assembled using pregap4, present in the Staden Package.^20^ All Brazilian *vir-e* sequences identified were submitted to GenBank (GenBank accession numbers:MZ357628-MZ357688).

Nucleotide and amino acid sequences were aligned and analysed using ClustalX.^21^ A dendrogram was created by ClustalX based on VIR-E amino acid sequences. Antigenicity of VIR-E sequences were analysed using VaxiJen predictor at threshold 0.5.^22^

*Cloning, recombinant expression and purification of P. vivax antigens* - One *vir*-E sequence was chosen to be recombinant expressed in bacterial system and from now on is denominated PvBrVIR-E. The sequence was cloned into pGEX-4T-3 and transformed into competent *Escherichia coli* STAR BL21 (DE3). The expression was inducted with 0.5mM isopropyl-β-D-1-thiogalactopyranoside (IPTG) at 18ºC, overnight. Bacterial cultures were then centrifuged at 6.000g for 10 min at 4ºC and the pellets were lysed using lysis buffer (10 mM Tris-HCl pH 8, 150 mM NaCl, 1 mM EDTA) with protease inhibitor (*cOmplete™, Mini, EDTA-free Protease Inhibitor Cocktail* Roche) and incubated at 4ºC for 1 h. Bacteria culture were than disrupted using an M-110L Pneumatic High Shear Fluids Processor (Microfluidcs). The soluble fraction was purified using *Glutathione Sepharose 4B* (GE Healthcare) following the manufacturer’s instructions. Protein expression was analysed by sodium dodecyl sulphate-polyacrylamide gel electrophoresis (SDS-PAGE) and western blot. Recombinant *P. vivax* apical membrane antigen 1 variant 5 (PvAMA1V5) and PvAMA1V16 were expressed and purified according as previously described.^23^

*Detection of naturally acquired antibodies to P. vivax antigens* - The presence of immunoglobulin G (IgG) against PvBrVIR-E, PvAMA1V5 and PvAMA1V16 and IgM to PvBrVIR-E recombinant proteins were measured in 80 plasma samples by in-house indirect enzyme-linked immunosorbent assay (ELISA) as previously described.^23^ The optical density (OD) was measured at 490 nm with wavelength correction at 595 nm using microplate reader (BioTek, Winooski, Vermont, USA). All plates had controls with anti-GST primary antibody (anti-GST control). Twenty plasma samples from healthy individuals from a nonendemic region were used as negative control. The absorbance values were normalised using OD values from anti-GST. The OD cut-off values were 1.0 for PvBrVIR-E IgM, 1.12 for PvBrVIR-E IgG, 0.8 for PvAMA1V5 and 0.85 for PvAMA1V16. Finally, the reactivity index (RI) was obtained from the ratio of absorbance value of each sample and the cut-off value (mean plus three standard deviation of the negative control absorbances). RIs > 1 were considered positive. Since the proteins were expressed with a GST tag, to avoid bias produced by a possible reactivity of the GST tag, absorbance values of recombinant GST against each plasma sample were evaluated and subtracted from absorbance values of each sample. The OD values to GST ranged from 0 to 0.4 to IgM and 0 to 0.55 to IgG antibodies.

*Binding assay of PvBrVIR-E to CHO-ICAM1* - Chinese hamster ovary (CHO) cells expressing constitutively ICAM1 were grown in RPMI 1640 medium (Sigma) and supplemented with 10% foetal bovine serum (FBS) and gentamicin, at 37ºC, in an atmosphere of 5% CO2. Cells were blocked with blocking solution [phosphate-buffered saline (PBS) solution containing 2% FBS] for 30 min, at 37ºC. Following the blockade, cells were incubated for 1 h at 37ºC with 10 µg of recombinant PvBrVIR-E or GST proteins. Subsequently, cells were washed three times with blocking solution and then the polyclonal α-GST (Sigma-Aldrich) produced in rabbit (dilution 1:200) was added to the wells. Cells were again washed three times with blocking solution and incubated with 1:100 anti-rabbit antibody conjugated to Alexa488 (Thermo Fisher Scientific). Next, cells were evaluated on Leica DMI6000 fluorescence microscopy.

*Statistical analyses* - To assess whether there were paired differences between antibody response to the recombinant proteins, Friedman test was applied, followed by pairwise comparisons using Nemenyi post-hoc test. Spearman’s correlation coefficient (rho) was estimated and the Spearman’s test was applied to inspect the relationship between PvBrVIR-E, PvAMA1V5 and PvAMA1V16 reactivity indexes and the following haematological parameters: platelets levels, total lymphocytes, neutrophils number of red blood cells, hematocrit and hemoglobin. Additionally, based on haematological parameters, two qualitative variables were derived: anaemia and thrombocytopenia, considered present, respectively, if hemoglobin levels was under 12 g/dL and if platelet count was lower than 150 × 10^3^ per μL. The reactivity index of each recombinant protein was compared between the categories (present or absent) of these variables through two sample Wilcoxon test. All tests were applied considering a 5% significance level.

*Ethics* - Ethical permission for the study was obtained by the local ethical committee in Manaus, Amazonas State, Brazil (CAAE-0044.0.114.000-11). Samples were collected after obtaining written consent from all individuals. After blood sampling, patients were treated for malaria according to national guidelines.

## RESULTS

*Genetic diversity of P. vivax vir-E* - *P. vivax vir*-E sequences were amplified from six Brazilian isolates. PCR fragments were cloned into a plasmid vector and sequenced. After quality control analyses and sequence assembly, a total of 61 *vir*-E sequences were obtained (median =11, range: 2-15 per isolate), with a size varying from 795 to 864 base pairs each [Supplementary data (Table I)]. The overall nucleotide identity between sequences was 82.55% (± 14.01). Out of these 61 sequences, 19 had a stop codon and were considered as pseudogenes. Among the remained ones, 38 were unique sequences. The overall amino acid identity between sequences was 79.73% (± 18.93). The antigenicity prediction was evaluated for the 38 amino acid sequences and 36 out of 38 were predicted to be antigenic (Vaxijen score > 0.5). Among the antigenic sequences, 7 had scores higher than 0.6 and were considered highly antigenic [Supplementary data (Table II)].

**Fig. 1:**
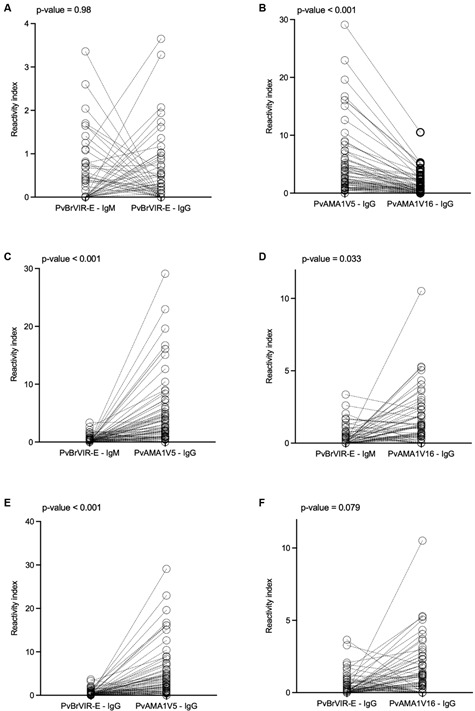
naturally acquired antibodies against *Plasmodium vivax* antigens. Human IgG and IgM antibody responses to PvBrVIR-E and IgG to PvAMA1 variants were detected in individuals infected with *P. vivax* (n = 80) and pairwise compared: reactivity index to PvBrVIR-E (A). Reactivity index to PvAMA1 variants (B). Reactivity index to PvBrVIR-E IgM compared to PvAMA1V5 (C) and PvAMA1V16 (D). Reactivity index to PvBrVIR-E IgG compared to PvAMA1V5 (E) and PvAMA1V16 (F). Significant differences were calculated by Friedman test, followed by pairwise comparisons using Nemenyi post-hoc test. p-values are indicating in the figures.

A phylogenetic tree was build based on amino acid sequences of all *vir*-E translated sequences [Supplementary data (Fig. 1)]. Thirty sequences shared more than 92% of amino acid identity (mean 97.15% ± 2.04). One of those antigenic sequence (Vaxijen score: 0.6172) here for denominated as PvBrVIR-E (GenBank accession number: MZ357651), was chosen as a representative sequence from VIR subfamily E and it was expressed and evaluated for antigenicity profile.

*Study subjects and haematological parameters* - A total of 80 infected individuals from Manaus were analysed. The haematological characteristics of the subjects are shown in Table. In this study, 29 (36.25%) individuals had hemoglobin levels below 12g/dL and were considered anaemic, while 64 (80%) individuals were thrombocytopenic (platelet count lower than 150 × 10^3^ per μL).

**TABLE:** Haematological data of *Plasmodium vivax* infected patients

Parameters	*P. vivax* patients n = 80
Hematocrit (%), median (IQR)	40.75, (36.2-43.3)
Hemoglobin (g/dL), median (IQR)	12.75, (11.3-13.8)
Anaemia, n (%)	29, (36.25)
RBC (10^6^/µL), median (IQR)	4.57, (4.05- 5.0)
Platelets (10^3^/µl), median (IQR)	89.5, (53.25-137.5)
Thrombocytopenia, n (%)	64, (80)
Neutrophils (%), median (IQR)	3.4, (2.3-4.5)
Lymphocytes (10^3^/µl), median (IQR)	0.9, (0.6-1.4)

Anaemia was considered when hemoglobin levels were under 12 g/dL. Thrombocytopenia was considered if platelet count was lower than 150 × 10^3^ per μL.

**Fig. 2:**
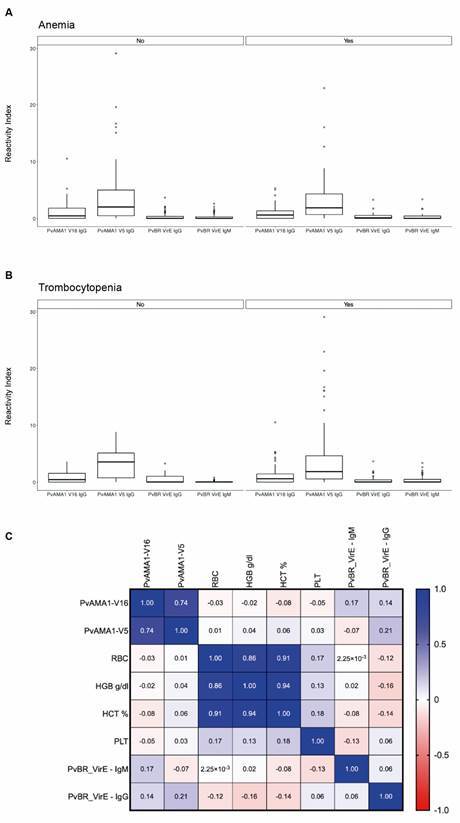
antibody response and their relationship with haematological parameters. Naturally acquired antibody response to PvBrVIR-E and PvAMA1variants and haematological parameters were evaluated in individuals infected with *Plasmodium vivax* (n = 80). (A) PvBrVIR-E and PvAMA1 reactivity index distribution in anaemic/ non-anaemic individuals. (B) PvBrVIR-E and PvAMA1 reactivity index distribution in individuals with/without thrombocytopenia. (C) Spearman’s correlation coefficient (rho) between antibody response and haematological parameters.

*Naturally acquired antibody to PvBrVIR-E* - To verify the antigenicity of PvBrVIR-E protein, the presence of naturally acquired IgG and IgM antibodies against this protein was evaluated. The purified recombinant PvBrVIR-E has a GST tag and an expected size of approximately 58 kDa (32kDa from rPvBrVIR-E itself and 26kDa from GST tag). The quality and purity of the recombinant protein was checked by western blotting [Supplementary data (Fig. 2)].

Naturally acquired IgM and IgG antibodies were evaluated in 80 patients infected with *P. vivax* from Manaus (Table). The majority of individuals had no detectable IgM or IgG antibodies to PvBrVIR-E (n = 61, 76.25%), while 19 individuals (23.75%) had IgM or IgG antibodies to PvBrVIR-E. The presence of IgM antibodies against PvBrVIR-E was detected in 12.5% (10/80) of *P. vivax* patients (Fig. 1A). The same frequency (12.5%, n = 10) was observed for the presence of anti-PvBrVIR-E IgG antibodies (Fig. 1A, Friedman test, followed by pairwise comparisons using Nemenyi post-hoc test, p = 0.98). Only one individual had IgM and IgG antibodies to PvBrVIR-E. The reactivity index values varied from 0 to 3.36 for IgM and 0 to 3.65 for IgG.

To evaluate if this low frequency of antibodies were specific against PvBrVIR-E antigen, the presence of IgG antibodies against two previously characterised PvAMA1 variants^23^ was also investigated. IgG antibodies were detected in 32 out of 80 (40%) individuals for PvAMA1V16 and 50 out of 80 (62.5%) individuals for PvAMA1V5. Twenty-seven individuals (33.75%) had no detectable IgG antibodies to any of those PvAMA1 variants (Fig. 1B).

Paired differences in reactivity index were observed between PvAMA1 variants, where the variant PvAMA1V5 was more frequently recognised than PvAMA1V16 (Fig. 1B, Nemenyi post-hoc test, p-value < 0.001). In a similar way, differences were observed between PvBrVIR-E IgM response and both PvAMA1 variants IgG response (Fig. 1C-D), and PvBrVIR-E IgG response and PvAMA1V5 IgG response (Fig. 1E). However, no difference was observed between PvBrVIR-E IgG and PvAMA1V16 IgG (Fig. 1F, Nemenyi post-hoc test, p-value = 0.079).

The presence of naturally acquired antibodies to PvBrVIR-E and PvAMA1 antigens were evaluated in anaemic/non-anaemic and thrombocytopenic/non-thrombocytopenic individuals. However, no differences were observed between those groups (Fig. 2A-B), as well as no correlation between haematological parameters and antibody response were observed (Fig. 2C).

**Fig. 3:**
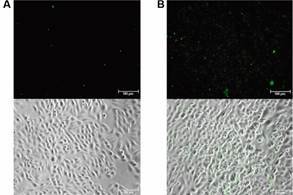
binding of PvBrVIR-E on CHO-ICAM1. Recombinant GST (A) or recombinant PvBrVIR-E (B) were incubated with CHO-ICAM1 cells and binding was detected by anti-GST on Leica DMI6000 fluorescence microscopy. Green spots represent the protein binding to CHO-ICAM1 cells detected by the fluorescent antibody.

*PvBrVIR-E binds to endothelial receptor ICAM-1* - Since VIR proteins are implicating to mediate the cytoadhere of *P. vivax* to the endothelial cells, the capacity of the recombinant PvBrVIR-E protein to adhere to CHO cells expressing ICAM1 was evaluated. The recombinant PvBrVIR-E was adherent to CHO-ICAM1 while it was not observed for the recombinant GST protein (Fig. 3).

## DISCUSSION

The major virulence antigens in *P. vivax* are the VIR proteins which are codified by genes from the variant *vir* superfamily. This family is extremely diverse and clustered in subgroups, suggesting distinct localisation and functions.^13^ ^,^ ^14^ ^,^ ^24^ Among all multigenic families described in *Plasmodium* species, *vir* genes are one of the most polymorphic.^25^ Among the *vir* subfamilies the *vir*-E group presents a PEXEL motif, suggesting that these proteins would reach the surface of the infected reticulocyte.^16^ Here, the genetic diversity of this subfamily, as well as its antigenicity and binding capacity were evaluated. When diversity of *vir*-E gene was assessed, 38 coding genes were identified in isolates. Despite the high variability of *vir* genes, a phylogenetic analysis showed a cluster of similar sequences whereas most of isolates here analysed had a representative gene, showing some degree of conservation among *P. vivax* Brazilian isolates. Even though almost 20% of the *P. vivax* genomes codifies VIR antigens, very little was explored about its role. The *vir* sequences here identified were majority coding sequences and predicted to be antigenic. However, a high percentage of truncate sequences were also found and considered pseudogenes. A high number of pseudogenes are frequently observed in multigenic gene families from *P. vivax* and *P. falciparum* field isolates.^16^ ^,^ ^26^

The host immune response against VIR antigens is not well characterised mainly due to its high polymorphism rates that difficult the amplification of coding sequences and expression of the recombinant proteins for immunoassay.^16^ Besides, the lack of a long-term *in vitro* culture for *P. vivax* is also an obstacle to the comprehension of immunological mechanisms.^27^ ^,^ ^28^ The expressive number of distinct *vir* genes on annotated genomes is also a challenge in the study of these antigens.^12^ Here, a *vir*-E gene identified from *P. vivax* field isolates from Brazil was recombinant expressed.

As previously observed, VIR proteins are antigenic, but the frequency of individuals that present antibodies against these antigens are quite heterogeneous, depending on the VIR antigen and the geographic region evaluated.^29^ ^,^ ^30^ Thus far, the frequency of individuals that had antibodies to recombinant VIR proteins are lower in Brazil when compared to Papua New Guine and Guatemala.^29^ Here, 12.5% of the study population had IgG antibodies to PvBrVIR-E while in a previous study with populations from Brazil identified antibodies in 26% and another one found between 1 to 42% of the population, depending on VIR antigen analysed. When comes specifically to the response to a VIR-E antigen the number of responders drops to 2.5-10%.^30^ The frequency of individuals that had IgM antibodies to PvBrVIR-E was similar for IgG (12.5%) and only one individual had the presence of both classes of antibodies. It is important to note that all individuals were living in malaria endemic area and therefore exposed to *P. vivax* infection. So far, there is no information about how is the dynamic of the antibody response to VIR antigens and how long they can last.

The low frequency of responders to VIR antigens is quite expected since the diversity of VIR antigens is high and it is still not known which of these antigens are going to be expressed by the parasite. Lesser is known about how long these antibodies could persistent or cross react between different VIR antigens.

Individuals exposed to *P. vivax* frequently have higher response to polymorphic merozoite antigens than to VIR antigens.^30^ ^,^ ^31^ In this study, despite the low frequency of VIR-E responders, most of individuals had antibodies to the microneme antigen PvAMA1 (66.25%). Due the sample limitation, only IgG antibodies to PvAMA1 variants were evaluated. Similar results were also observed when compared the response to VIR-E antigen and PvAMA1 in different regions from North of Brazil.^30^ This difference is most likely due high frequency of polymorphisms found at VIR antigens compared to other polymorphic antigens.^25^

Recently, we showed that PvAMA1V5 was more frequently recognised than PvAMA1V16 in populations from distinct locations from North of Brazil.^23^ Here, similar results were observed (Fig. 1B), confirming that PvAMA1V5 is more frequently recognised in North of Brazil. The sequence coding PvAMA1V5 was also more frequently identified in a population from Manaus and several polymorphic sites between PvAMA1V5 and PvAMA1V16 were described.^23^

In fact, polymorphisms can have a direct impact on the protein structure and therefore, on the B cell epitope. So far, the crystal structure of VIR antigens has not been solved and since these antigens are very polymorphic is difficult to model them based to any other known antigens. Although there are more tools to predict antigenicity, it is relevant to point out that Vaxijen2.0, different from other tools to predict antigenicity, used not only bacteria and virus sequences to develop it, but also parasites proteins yields trustworthy results.^22^ In contrast, Vaxign2, Vaxign-ML and Vaxijen3.0 although developed as a machine learning (ML) model; which has better results; their training sets were exclusively with bacterial proteins.^32^ ^,^ ^33^ ^,^ ^34^ Therefore, they are not to be trusted in regards of antigenicity of a protozoa parasite. Dalsass and colleagues noted that a satisfactory way of predicting antigenicity would be to combine the results of a filtering method, such as Vaxijen2.0, with another using ML.^35^ Unfortunately, there are no ML based predictors trained with protozoa data, so our most truthfully results are those of Vaxijen2.0.

Few studies have been explored the function of VIR proteins. In part, this lack of data is explained by the difficulties while working with *P. vivax*. As recently observed in several studies, *P. vivax* can adhere to endothelial receptors and form rosettes in a similar fashion as *P. falciparum*.^36^ ^,^ ^37^ However, unlike *P. falciparum*, PfEMP1 and RIFIN antigens are absent in *P. vivax*. Therefore, some other antigens must be implicated in the adhesion phenomena. In fact, a VIR antigen from subfamily C have been demonstrated to adhere to ICAM-1.^15^ Here, the adhesion to the PvBrVIR-E antigen to CHO-ICAM1 was also observed, suggesting that this subfamily, as for subfamily C, can be implicated in adhesion phenomenon. Nevertheless, extracellular vesicles derived from plasma of *P. vivax* infected patient were taken up by human spleen fibroblast and upregulated the expression of ICAM-1.^38^

Taking all together, here we showed that *vir*-E subfamily codifies antigenic VIR-E protein and it is recognised by a small part of *P. vivax* infected individuals. Nonetheless, the recombinant PvBrVIR-E was also adherent to CHO-ICAM1 cells. These data open avenues for the study of variant antigens in *P. vivax* and their implications on immunity and adhesion.
