# Supplementary material for: Antigenicity and adhesiveness of a Plasmodium vivax VIR-E protein from Brazilian isolates
Source: Mem Inst Oswaldo Cruz. 2022 Feb 4;116:e210227. doi: 10.1590/0074-02760210227 (PMC8824159; doi:10.1590/0074-02760210227)
Supplement: Supplementary file 1 [file 1678-8060-mioc-116-e210227-s.pdf]

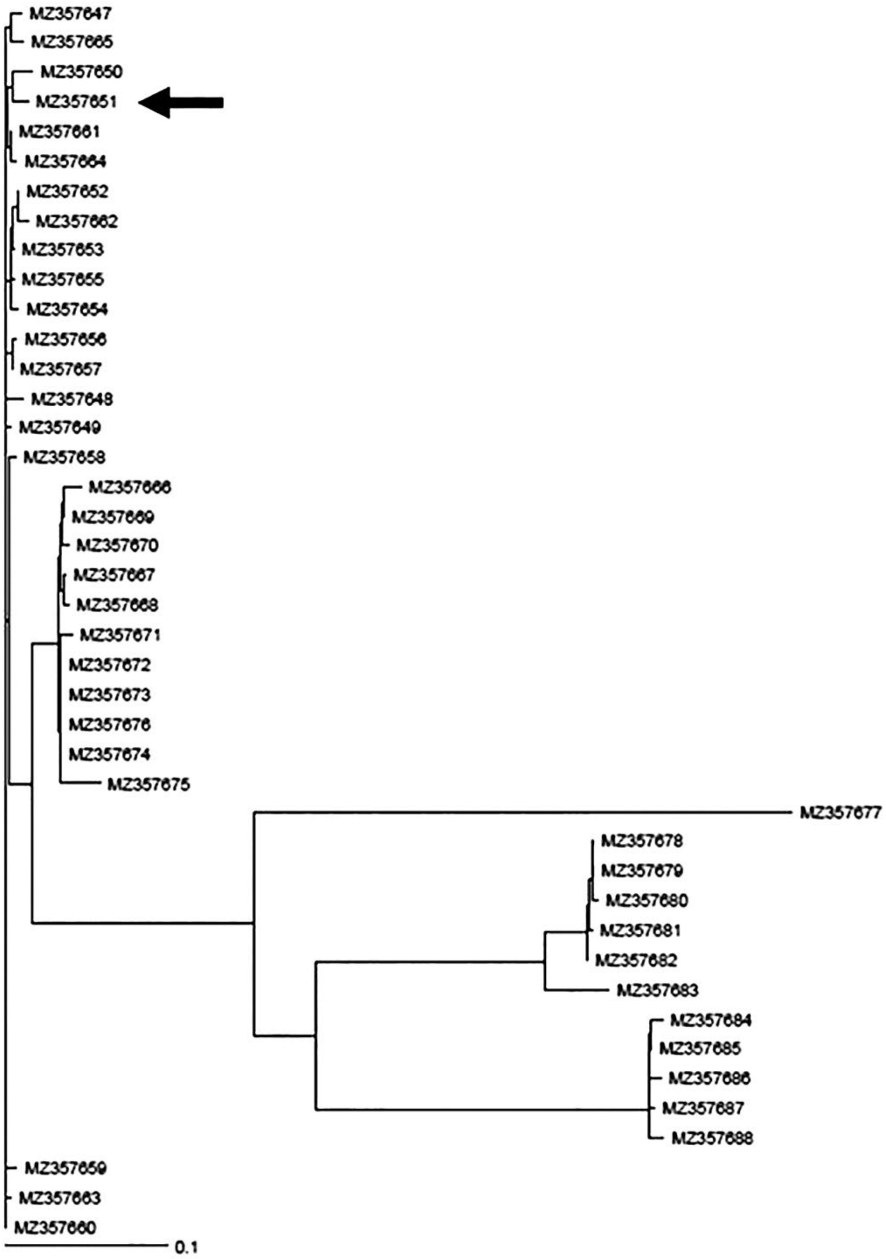

Fig. 1: sequence analysis. Dendrogram of phylogenetic relationships between *Plasmodium vivax vir*-E sequences from Brazilian isolates. The arrow indicates the sequences that were chosen to be expressed.

CC BY

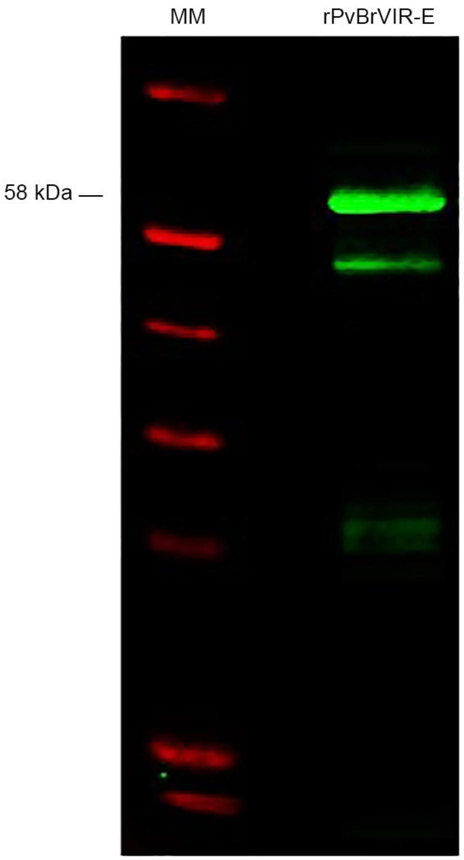

Fig. 2: western blotting of recombinant PvBrVIR-E. Anti-GST antibody recognized recombinant PvBrVIR-E protein at ~58kDa, detected by fluorescence using Odyssey® CLx Imaging System (LI-COR Biosciences). MM: molecular marker.

TABLE I

Information of *vir*-E sequences from Brazilian *Plasmodium vivax* isolates. *Vir*-E genes were amplified from 6 *P. vivax* isolates. Number of sequences reads; unique sequences and pseudogenes are indicated

Isolate ID	Sequence reads	Unique sequences	Pseudogenes
Pv11-89	2	2	1
Pv11-101	14	11	4
Pv12-21	15	9	4
Pv12-29	13	7	6
Pv12-73	9	5	3
Pv12-80	8	4	1
Total	61	38	19

TABLE II

Predicted antigenic sequences. Antigenic analysis of translated vir-E sequences was evaluated by Vaxijen Server. Scores higher than 0.5 are predicted to be antigenic. In bold the sequence that was recombinant expressed

Sequence ID	Vaxijen score
MZ357688	0.6300
MZ357687	0.6184
**MZ357651**	**0.6172**
MZ357685	0.6168
MZ357686	0.6151
MZ357684	0.6094
MZ357677	0.6013
MZ357656	0.5976
MZ357650	0.5939
MZ357652	0.5920
MZ357655	0.5915
MZ357647	0.5846
MZ357654	0.5815
MZ357653	0.5769
MZ357663	0.5701
MZ357648	0.5665
MZ357658	0.5635
MZ357664	0.5619
MZ357662	0.5606
MZ357660	0.5599
MZ357649	0.5596
MZ357657	0.5593
MZ357675	0.5579
MZ357661	0.5534
MZ357682	0.5533
MZ357659	0.5498
MZ357678	0.5494
MZ357679	0.5494
MZ357668	0.5491
MZ357681	0.5475
MZ357667	0.5474
MZ357671	0.5467
MZ357669	0.5456
MZ357672	0.5439
MZ357673	0.5439
MZ357674	0.5439
MZ357676	0.5439
MZ357680	0.5436
MZ357665	0.5404
MZ357670	0.5391
MZ357683	0.4953
MZ357666	0.4886
